# Handwriting in primary school: comparing standardized tests and evaluating impact of grapho-motor parameters

**DOI:** 10.1007/s11145-024-10562-3

**Published:** 2024-06-20

**Authors:** Laura Sparaci, Valentina Fantasia, Chiara Bonsignori, Cecilia Provenzale, Domenico Formica, Fabrizio Taffoni

**Affiliations:** 1 Institute of Cognitive Sciences and Technologies (ISTC), National Research Council (CNR) of Italy, Via Nomentana 56, 00161 Rome, Italy; 2 Department of Philosophy and Cognitive Science, Faculties of the Humanities and Theology, Lund University, Lund, Sweden; 3 Advanced Robotics and Human Centered Technologies –CREO Lab, University Campus Bio-Medico di Roma, Rome, Italy; 4 School of Engineering, Newcastle University, Newcastle upon Tyne, UK

**Keywords:** Children, Grapho-motor skills, Cursive handwriting, Grapho-motor parameters, Dysgraphia, Primary school

## Abstract

A growing number of primary school students experience difficulties with grapho-motor skills involved in handwriting, which impact both form and content of their texts. Therefore, it is important to assess and monitor handwriting skills in primary school via standardized tests and detect specific grapho-motor parameters (GMPs) which impact handwriting legibility. Multiple standardized tools are available to assess grapho-motor skills in primary school, yet little is known on between-test agreement, on impact of specific GMPs on children’s overall performance and on which GMPs may be specifically hard to tackle for children that are starting to consolidate their handwriting skills. These data would be extremely relevant for clinicians, therapists and educators, who have to choose among different assessment tools as well as design tailored intervention strategies to reach adequate performance on different GMPs in cases of poor handwriting. To gain better understanding of currently available standardized tools, we compared overall performance of 39 Italian primary school children (19 second graders and 20 third graders) experiencing difficulties with handwriting on three standardized tests for grapho-motor skills assessment and explored the impact of individual GMPs on child performance. Results showed some agreement between tests considering all children in our sample, but no agreement in second grade and only limited agreement in third grade. Data also allowed highlighting significant correlations between some GMP scores and children’s overall performance in our sample. Finally, children in our sample appeared to experience specific difficulties with some GMPs, such as letter joins and alignment.

## Introduction

Even if keyboards are extensively used for writing both at home and in work environments, we rely on writing by hand using a pen or a stylus in multiple occasions (Santangelo & Graham, 2016). In fact, traditional handwriting with pen and paper has been gradually supplemented also by contemporary technologies allowing screen-based handwriting with styluses (e.g., phone, tablets, smart notebooks), also leading researchers to reconsider handwriting skills and their impact on learning (Ihara et al., 2021; Karavanidou, 2017; Osugi et al., 2019). For example, Ose Askvik and colleagues (2020) investigated which activity between handwriting, typing or drawing would best support learning in the classroom using high-density EEG to measure brain activity during different tasks in a group of eleven-year-olds. Children were presented with 15 words of varying difficulty and asked to either type, write in cursive handwriting or draw the presented word using a keyboard or a stylus on a screen. Both cursive handwriting and drawing, in contrast to typing, led to event-related synchronized activity in the theta range in temporal parietal and central brain regions, associated to positive effects on memory and encoding of novel information, thus suggesting that these activities may lead to better conditions for learning (Ose Askvik et al., 2020). Other studies suggest that movements involved in handwriting may allow greater memorization of new letters and words in primary school children (Ihara et al., 2021; Longcamp et al., 2005).

Handwriting is still a prominent activity throughout primary school, where children dedicate, on average, 30–60% of their time to fine-motor tasks; of which 85% are paper-and-pencil based and involve grapho-motor skills (copying text from the board, repetitive writing, writing from dictation, creative writing, drawing, etc.) (Cutler & Graham, 2008; McHale & Cermak, 1992). Given the importance of grapho-motor skills involved in handwriting for learning, it is important to ensure that primary school students develop fluent and legible handwriting. Santangelo and Graham (2016) argue that acquiring fluent and legible handwriting has an effect both on a student’s presentation (*presentation effect*) and on her writing (*writer effect)*. The *presentation effect* refers to the fact that a text’s legibility (i.e., its form) may influence others’ judgments about the actual quality of the ideas it expresses (i.e., its contents) (Graham et al., 2011). In fact, multiple studies show that texts written in poor handwriting will negatively bias teachers’ evaluations (Chase, 1986; Greifeneder et al., 2010; Klein & Taub, 2005; Sweedler-Brown, 1992). The *writer effect* is an overarching term used instead to group multiple ways in which poor handwriting effects the writer himself both while writing and afterwards. For example, research shows that poor grapho-motor skills and handwriting speed influence the ability to keep up with thoughts and ideas, while difficulties in fine-motor planning have an impact on attentional demands during writing tasks (Berninger et al., 1992; Berninger, 1999; Graham, 1990; Graham et al., 2000; Jones & Christensen, 1999; Skar et al., 2022; Tseng & Cermak, 1993). Furthermore, poor grapho-motor skills also impact an individual’s lifelong propensity to write (Santangelo & Graham, 2016). For example, children with poor grapho-motor skills have difficulties keeping up with dictation or other handwritten work required in primary school and may attempt to avoid writing whenever possible developing a mindset that they cannot write, which is hard to overcome even in adulthood (Feder & Majnemer, 2007; Santangelo & Graham, 2016).

Both *presentation* and *writer effect* testify that it is important for students to acquire good grapho-motor skills (Graham et al., 2011). However, to date, numerous students struggle with the acquisition of fluid and legible handwriting in primary school (Marquardt et al., 2016) often resulting in different levels of difficulty with handwriting (Lopez & Vaivre-Douret, 2023) ranging from mild (children classified as ‘poor writers’) to severe (children with ‘dysgraphia’). Boys often experience more difficulties than girls, suggesting the need for more tailored interventions (Marquardt et al., 2016; Maurer, 2023). In the eighties and nineties, estimated percentage of elementary-school children experiencing handwriting difficulties was 10–21% in the Netherlands, UK and Norway (Alston, 1985; Hulstijn & Mulder, 1985; Maeland, 1992; Rubin & Henderson, 1982; Smits-Engelsman et al., 1995). In 2015, a German survey on handwriting skills in primary and secondary schools involving 1907 teachers, showed that 30% of girls and 50% of boys were judged as ‘poor writers’ by their teachers (Marquardt et al., 2016). In the same year a teacher survey on 2090 children in India’s Telangana State considering fourth- and fifth-graders in 30 private schools, showed 15% incidence of dysgraphia (Indira & Vijayan, 2015). In 2022 governmental data on more than 6 million primary, middle and high school students in public and private schools in Italy, reported that 1.4% had a dysgraphia diagnosis, and comparison with previously collected data showed that cases of dysgraphia went from over 30 K to over 90 K in 7 years MI–DGSIS (2022). While percentages largely differ across nations, mainly due to differences in assessment methods and diagnostic criteria, numbers and increase in children with difficulties suggest the need to monitor handwriting acquisition and reach better understanding of underlying mechanisms responsible for scarce legibility assessments in primary school (Vaivre-Douret et al., 2021).

To date, children with difficulties in grapho-motor skills are usually formally assessed relying on cursive handwriting and standardized pen-and-paper tests starting from second-grade.^1^ Attempts have been made at building software tools for grapho-motor assessments, but they are still mostly used for experimental purposes and, in many countries, lack normative data (Asselborn et al., 2020; Capellini et al., 2020; Dimauro et al., 2020; Falk et al., 2011; Mekyska et al., 2017; Provenzale et al., 2022, 2023). Clinical referral usually starts with teachers’ reports to parents based on observations of child performance in class. Clinical evaluation is most commonly based on observation of notebooks and assessment of grapho-motor skills via a chosen standardized test, which allows post-hoc analysis of handwritten samples to extract: overall handwriting performance and, in some cases, individual scores on selected grapho-motor parameters (GMPs) (Rosenblum et al., 2003). GMPs contribute to text legibility and involve measures such as: letter form, size, alignment, spacing, etc. (Rosenblum et al., 2003).^2^

In Italy there are three standardized tests for grapho-motor skills assessment in primary school: the Italian *Batteria per la Valutazione della Scrittura e della Competenza Ortografica* (BVSCO; Tressoldi et al., 2019), the Italian standardized version of the *Brave Handwriting Kinder* (BHK; Brina et al., 2010) and the Italian *Test per la Valutazione delle Difficoltà Grafo-Motorie e Posturali della Scrittura* (DGM-P; Borean, 2012). These tests allow the assessment of the overall handwriting and GMPs performance from a written sample, but they differ in number, type and methods used (e.g., the BVSCO only measures handwriting speed, while the BHK measures 13 GMPs and the DGM-P 12 GMPs) (see below for a more detailed description). The BHK test has been shown to correlate well with tests measuring visuo-motor and fine-motor skills (Duiser et al., 2014; Kaiser et al., 2009; Volman et al., 2006) and the DGM-P has shown good agreement with visual-spatial skills assessments (Scordella et al., 2015). However, very limited data is available on agreement between these tests on children’s overall handwriting performance. In fact, we found only one descriptive study comparing overall handwriting performance of 35 Italian third-grade students on the BHK and DGM-P tests. This study highlighted scarce agreement between tests, also suggesting that the DGM-P test tended to pinpoint grapho-motor difficulties in many children that were not detected by the BHK test (Neri et al., 2017). However, this study is only descriptive providing no statistical data.

Information on between-test agreement is relevant for clinicians, who need to choose among different standardized tools to assess handwriting skills, as well as for occupational therapists, using these tools to monitor effectiveness of specific intervention strategies. More relevantly, GMPs measured by standardized tests impact legibility across multiple dimensions and concur to poor handwriting and dysgraphia, also providing an understanding of a child’s profile of strengths and weaknesses, which may be used in planning treatment and exercises (Hamstra-Bletz & Blöte, 1993; Volman et al., 2006). For example, if a child shows inadequate performance on a specific GMP such as letter alignment (e.g., producing a text in which entire words or single letters often ‘float’ below or above the line), use of dedicated notebooks with highlighted lines to delimit writing space may be suggested to teachers or parents (Pellegrini & Dongilli, 2010). While it is well known that overall legibility scores are determined by GMPs, we still do not know whether lower performance on specific GMPs is associated with worse overall handwriting performance. We also do not know whether specific GMPs prove particularly difficult for primary school children with poor handwriting.

Therefore, the aim of the present study was answering three main research questions. The first research question was: is there agreement between tests for grapho-motor skills assessment in primary school children that are consolidating their grapho-motor skills (i.e., children in second and third grade)? To answer this question, we assessed grapho-motor skills in 39 primary school children (in second and third grade) using the three standardized tests most commonly used in Italy (i.e., BVSCO, BHK, DGM-P). Then we compared overall handwriting performance in the three tests in our entire sample (to evaluate level of agreement between tests) and in second and third grade respectively (to measure consistency in test agreement). Considering our entire sample, we expected to find at least some agreement between these commonly used tests for grapho-motor skills assessment, given that they are all analytic tools relying on GMPs. Considering only third-graders and based on the limited literature available (Neri et al., 2017), we expected to find no agreement between the BHK and the DGM-P tests in third grade. No predictions were made on test agreement in relation to second-graders as no dedicated literature was available.

Our second research question was to investigate whether poor performance on specific GMPs was associated with worse overall handwriting performance in primary school children. To answer this point, we analyzed presence of a positive item-rest correlation between individual GMPs and overall handwriting performance respectively within the BHK and the DGM-P tests in our entire sample. This analysis was aimed at pinpointing which GMPs may be mainly and significantly associated with a child’s overall score in each test. We expected that some characteristics of handwriting (as measured by specific GMPs) would emerge as more relevant towards children’s overall performance, given that based on teachers’ questionnaires some aspects of cursive handwriting are often reported as harder to master for children in primary school (Marquardt et al., 2016), but no predictions were made on which ones.

Finally, our third research question was to explore whether children experiencing difficulties with handwriting would show inadequate performance on specific GMPs. To address this point we analyzed the occurrence frequencies of children’s inadequate performances on GMPs in our entire sample (considering the BHK and the DGM-P tests). Some studies have attempted to build a taxonomy of common handwriting errors, often aiming to develop appropriate intervention strategies (Chandra et al., 2017), but little is known on whether specific GMPs assessed in standardized tests prove particularly hard to tackle for children experiencing handwriting difficulties. Given that this point has not been previously investigated relying on these tests, we could not make specific predictions on which GMPs would lead to more inadequate performances and considered this third research question mainly exploratory.

## Methods

### Participants

Children in our sample were part of a larger study investigating early communicative development and were all enrolled in a public primary school “Istituto Comprensivo Borgoncini Duca” in Rome, Italy (Sparaci et al., 2022). For sample participants to reflect difficulties in handwriting reported by teachers in the literature (Marquardt et al., 2016), as done by previous studies we asked educators to select three classes in which screening of handwriting skills would be appropriate (Sudsawad et al., 2001). Accordingly, three classes were selected by the school (i.e., 67 children), these classes were selected by the teachers’ board among all the ones present in the school because they believed them to include some poor writers. Parent consent was then obtained for a total of 39 children, which included 19 second graders (11 females and 8 males, mean chronological age 7.86 years) and 20 third graders (13 males and 7 females, mean chronological age 8.72 years). Parents were also asked to fill in a voluntary questionnaire to provide information on their children and on sample demographics. All parents complied with this request with the exception of 9 parents that chose not to fill in the questionnaire and one parent that chose not to fill in the information on current occupation. Sample demographics are summarized in Table 1. According to information from parent questionnaires all children were primary speakers of Italian and 5 children were exposed to a second language outside the school environment (4 children to English, 1 to Spanish). Seven children were born pre-term, but none had a previous history of language and/or learning disabilities, with the exception of 3 children who presented respectively mild phonological disorder, selective mutism and neuromotor delay when younger, overcome through therapy.

**Table 1:** Sample demographics

Demographics	Number of parents
*Maternal education*	
College degree	14
High school	15
Middle school	1
*Paternal education*	
College degree	14
High school	13
Middle school	3
*Maternal occupation*	
Office worker	15
Self-employed	7
Unemployed	3
Manager	2
Labourer	2
*Paternal occupation*	
Office worker	16
Self-employed	8
Unemployed	2
Manager	2
Labourer	1

Intellectual functioning (IQ) was directly assessed, for the purpose of the present study, in all children using Raven’s Colored Progressive Matrices (RCPM, Raven, 1962), and all children had an IQ of 90 or above (participants characteristics are summarized in Table 2). We also annotated vision impairments and handedness during the child assessments. Accordingly, all participants had normal or corrected to normal vision (3 children in our sample wore glasses) and they were all right-handed with the exception of 3 children in second grade and 3 children in third grade who were left-handed. The Ethical Committee of the Institute of Cognitive Sciences and Technologies (ISTC), of the National Research Council (CNR) of Italy approved all study purposes and procedures, which were then presented and explained to teachers as well as parents of participants in a dedicated meeting, requiring informed written consent prior to data collection.

**Table 2:** Participant characteristics

	Entire sample	Second grade	Third grade
Number	39	19	20
Gender	18 (F) 21 (M)	11 (F) 8 (M)	7 (F) 13 (M)
Handedness	33 (R) 6 (L)	16 (R) 3 (L)	17 (R) 3 (L)
Chronological age (months)	99.55 (6.20) 86.80–109.80*	94.26 (3.73) 86.80–99.3*	104.56 (3.02) 99.70–109.80*
IQ (RCPM)	108.72 (12.81) 90–130*	103.68 (9.55) 90–120*	113.50 (13.87) 90–130*

^*^Mean (SD) range

*RCPM* Raven’s colored progressive matrices

### Procedure

Children were individually assessed by dedicated research personnel in a quiet room at school. They were asked to sit at a school desk using their preferred chair and three gold-standard tests were used to assess grapho-motor skills in cursive handwriting: the Italian *Batteria per la Valutazione della Scrittura e della Competenza Ortografica* (BVSCO, Tressoldi et al., 2019), the Italian standardized version of the *Concise Assessment Scale for Children’s Handwriting* (*Brave Handwriting Kinder*) (BHK, Brina et al., 2010) and the Italian *Test per la Valutazione delle Difficoltà Grafo-Motorie e Posturali della Scrittura* (DGM-P, Borean, 2012). Order in which tests were administered was randomized. All tests are pen and paper assessments and required children to write a short text in cursive handwriting, which was later scored by second and first authors (respectively first and second coder).

### Materials

**Fig. 1:**
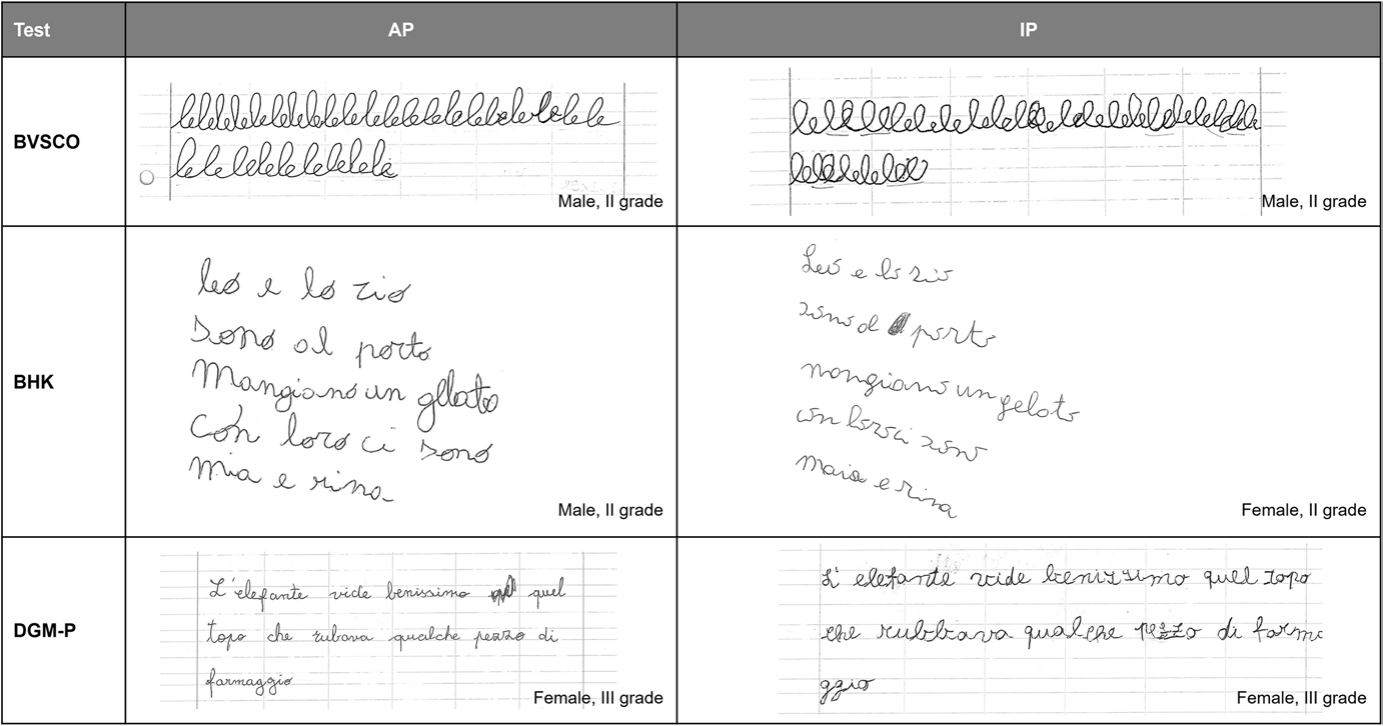
Child handwritten samples from the BVSCO, BHK and DGM-P tests respectively

The BVSCO (Tressoldi et al., 2019) is an Italian standardized test allowing to assess handwriting and orthographic competencies in children between first and fifth grade. The test is divided in 3 sub-sections: dictation, creative writing and handwriting velocity. For the scope of the present paper we considered only the BVSCO subtest dedicated to grapho-motor skills assessment (i.e., the cursive handwriting velocity subtest). Children were asked to observe an adult repeatedly writing the syllable “le” in cursive handwriting on a sheet of ruled paper (of the type most commonly used by the child) and then write this syllable themselves as many times as they could within one minute (see Fig. 1 for a child’s handwritten sample). Following test scoring procedures handwritten texts were used to assess handwriting *speed* (i.e., the only GMP measured in the BVSCO test) by counting the number of clearly recognizable/distinguishable graphemes produced by each child (*grapheme*_*child*_) and comparing this number to normative sample (NS) data from children in the same grade and trimester. This comparison allows to map child’s handwriting performance on two nominal levels (‘adequate’, ‘inadequate’) according to the following inequality:

$$graphme_{child} < \overline{grapheme}_{NS,class} - 2\sigma_{NS,class}$$

where ${\overline{grapheme} }_{NS,class}$ and ${\sigma }_{NS,class}$ are respectively the mean and standard deviation in number of graphemes produced in the NS group in the same class and trimester.^3^ If the inequality is satisfied, the child’s overall handwriting performance is considered ‘inadequate’; ‘adequate’ otherwise (see also Tressoldi et al., 2019 for detailed normative data).

The BHK test is the Italian standardized version of the original Dutch “Beknoptebeoordelingsmethode voor kinderhandschriften: BHK” (Hamstra-Bletz et al., 1987) assessing presence/absence of dysgraphia in children between second and fifth grade (Di Brina & Rossini, 2010; Loizzo et al., 2023). In the BHK children are asked to copy within five minutes as much as they can of a 28-line text shown to them on a printed sheet and composed of phrases with progressive level of difficulty. Children should copy the text in cursive handwriting on a white unlined paper using their usual style and pace. The entire handwritten sample is used to assess handwriting *speed*, which is obtained relying on NS according to the following equation:

1 $$Speed = \frac{{{{\text{char}}}_{{\text{child}}} - {{\overline{\text{char}}}}_{{{\text{NS,class}}}} }}{{\sigma_{{\text{NS,class}}} }}$$

where ${\overline{char} }_{NS,class}$ and ${\sigma }_{NS, class}$ are respectively the average number and the  standard deviation in the number of characters for the NS data referred to the same class of the child being evaluated, while the ${char}_{child}$ is the effective number of characters produced by the child.

The first five lines of handwritten text (see Fig. 1 for a child’s handwritten sample) are used to assess 13 GMPs: (1) *handwriting size*, (2) *margin alignment*, (3) *sentence alignment*, (4) *word spacing*, (5) *acute/long joins*, (6) *interrupted/overlapping joins*, (7) *letter collisions*, (8) *irregular letter size*, (9) *incoherent letter size*, (10) *letter distortions*, (11) *ambiguous letters*, (12) *self-corrections*, (13) *unsteady trace* (see Table 3 for a detailed description). *Handwriting size* and *margin alignment* are scored observing the first five lines of text as a whole, while all other GMPs are assessed on individual lines of text. Each GMP may obtain a score ranging between 0 and 5, except for GMP n 9 which ranges between 0 and 4 (higher scores indicating worse performance). Children’s overall performance score (OPS) is obtained by summing the 13 GMP scores, with an OPS score range between 0 and 64 (higher scores indicating worse performance). The BHK remaps child’s OPS (*OPS*_*child*_) with respect to NS according to the following equation:

$$OPS^{\prime} = \frac{{\overline{OPS}}_{NS,G} - {OPS}_{child}}{{\sigma_{NS,G} }}$$

where $\overline{OPS}_{NS,G}$ and *σ*_*NS,G*_ are respectively the average and standard deviation of the OPS measured for the NS data considering the same gender of the child.^4^ The handwriting is judged ‘inadequate’, or dysgraphic, when OPS’ ≤ − 1.5; in all other cases it is considered ‘adequate’ (Di Brina & Rossini, 2010; Loizzo et al., 2023).

**Table 3:** Detailed description of scoring criteria for individual GMPs as measured by the BHK test

GMP	BHK	Scoring
1	Handwriting size	Average handwriting size on the first 5 lines of text is measured in mm to evaluate whether handwriting is too large or too small and an overall score between 0 and 5 is given based on fixed test parameters based on this measurement and on the students’ class (e.g., a III grade student will obtain 0 if his/her handwriting is smaller or equivalent to 3 mm and a score of 5 if it greater or equivalent to 5 mm)
2	Margin alignment	Widening of the left-hand margin across the first 5 lines of text is measured according to a template showing different angles to evaluate correct/incorrect alignment and an overall score between 0 and 5 is given based on the template (e.g., a text completely aligned with the margin will receive a 0, a text showing a 20° angle with respect to the margin will receive a score of 5)
3	Sentence alignment	Vertical variance of letters within each line of text is evaluated based on a template straight line aligned with the first and last letter of the phrase. If one or more letters within 1 line of text float below or above the line a score of 1 is given, 0 is given in all other cases
4	Word spacing	Spacing between words in a line of text is deemed insufficient when it is inferior to the width of the letter “o” as written by the child. Spacing between words on each line of text is measured against this letter and if one or more words appear closer than the estimated letter size a score of 1 is given, 0 is given in all other cases
5	Acute/long joins	Presence of at least one acute turn and/or of an excessively long trait in connecting letters in each line of text is evaluated, if present 1 is given, 0 is given in all other cases
6	Interrupted/overlapping joins	Presence of at least one break and/or of absence of trace between letters in each line of text is analyzed, if present 1 is given, 0 is given in all other cases
7	Letter collisions	Presence of at least one collision and/or partial overlap between letters in each line of text is analyzed, if present 1 is given, 0 is given in all other cases
8	Irregular letter size	For each line of text, the height of the smallest and of the largest letter is measured using a template in mm. The ratio between these measures is then compared to the average ratios based on fixed parameters provided in the BHK test scoring manual. If the observed ratio is within the average ratio a score of 0 is given, 1 is given in all other cases (e.g., if the smallest letter on a line is 2 mm high the largest letter must not exceed 3 mm in height)
9	Incoherent letter size	In each line of text, the height of letters with extension (e.g., l, g, p) is compared to the height of letters without extensions (e.g., a, e, o). If letters with extensions do not differ at all or differ only minimally from letters without extensions a score of 1 is given, 0 is given in all other cases
10	Letter distortions	Each line of text is observed to detect presence of letters that do not respect the canonical shapes used in cursive handwriting (e.g., an “o” that is not a closed circle or a “t” with an eyelet). If present 1 is given, 0 is given in all other cases
11	Ambiguous letters	Each line of text is observed to detect presence of letters with a shape that may be confusing for the reader (e.g., an “i” that looks like and “e” or an “a” that may be confused with an “o”). If present 1 is given, 0 is given in all other cases
12	Self-corrections	For each line of text presence of letters that have been re-traced or re-written by the student is analyzed. If present 1 is given, 0 is given in all other cases
13	Unsteady trace	For each line of text presence of an unsteady or wiggly letter trace is observed. If present 1 is given, 0 is given in all other cases

The DGM-P is an Italian standardized test allowing to assess handwriting legibility in children between second and fifth grade (Borean, 2012). In the DGM-P test, children are asked to read a single phrase (containing all letters of the Italian alphabet) on a printed card and then copy it on a sheet of ruled paper (of the type most commonly used by the child) in cursive handwriting in two different conditions: using their best handwriting quality (best condition) and as fast as they can (fast condition). For the purpose of the present paper we only considered child performance in the best condition (see Fig. 1 for a child’s handwritten sample). Written texts are scored to assess 12 GMPs: (1) *speed*; (2) *letter forming*; (3) *self-corrections*; (4) *letter alignment*; (5) *letter distortions, interrupted/overlapping joins*; (6) *ambiguous letters*; (7) *incorrect letter size*; (8) *unrecognizable letters*; (9) *letter collisions*; (10) *max amplitude of letter misalignment*; (11) *max variation in size of letters without extension*; (12) *max variation in size of letters with extension* (see Table 4 for a detailed description). Best condition was timed to calculate GMP n 1 *speed*, measured as number of letters per seconds (including letters that the child may have incorrectly added to the text and letters that are completely erased and/or rewritten). Given that in the DGM-P test scores are calculated mostly by assessing individual letters written by children (rather than by assessing individual phrases, as in the BHK test), this test often requires consistent training in order to reach inter-coder reliability. All GMPs are individually compared to normative data as provided in the scoring manual to evaluate if performance on a given GMP is ‘adequate’, ‘at risk’ or ‘inadequate’. The number of ‘inadequate’ and ‘at risk’ GMPs (excluding speed and letter forming) is considered for the child’s overall performance assessment. In particular, the handwriting is judged ‘inadequate’ if a child shows a number of ‘inadequate’ GMPs ≥ 3, otherwise, the number of ‘at risk’ scores will be considered and compared to normative data to judge whether her performance is ‘adequate’, ‘at risk’ or ‘inadequate’ according to tables provided in test instructions (Borean, 2012, p. 76 for detailed tables).

**Table 4:** Detailed description of scoring criteria for individual GMPs as measured by the DGM-P test

GMP	DGM-P	Scoring
1	Speed	Speed is calculated by dividing the number of letters (including letters that the child may have incorrectly added to the text and letters that are completely erased and rewritten) by seconds taken to write the phrase
2	Letter forming	For each letter, consistent mistakes related to handwriting processes are evaluated. Specifically, a letter is considered incorrect in at least one of the following occurs: individual letter segments were traced in an incorrect order; horizontal letter segments were traced from left to right; vertical letter segments were traced from top to bottom; round letters were traced counter-clockwise; ascending eyelets were traced counter-clockwise; descending eyelets were traced clockwise; inefficient or discontinuous joins are present in reshaping joins (e.g., “ba”,“vi”,“zz”). Score reports number of incorrect letters
3	Self-corrections	Number of letters that have been erased, completely or partially rewritten or re-traced/adjusted is counted
4	Letter alignment	Vertical variance of single letters is measured using graph paper aligned with the ruled paper line. Each letter whose distance above or below the ruled paper line exceeds 1.5 mm is marked as misaligned. Score reports number of misaligned letters
5	Letter distortions, interrupted/overlapping joins	Number of letters that display at least one of the following: letter distortions (i.e., missing or interrupted letter trace; overlap in letter trace; lack of joins between each letter segments; unnecessary eyelets; eyelets traced as single lines) or interrupted/overlapping joins with adjacent letters
6	Ambiguous letters	Number of letters with a shape that may be confusing for the reader (e.g., an “i” that looks like and “e” or an “a” that may be confused with an “o”)
7	Incorrect letter size	Letters with extension (e.g., l, g, p) are measured using a graph paper template. If the extension is smaller than the letter body, the letter is counted as incorrect. Score reflects number of incorrect letters
8	Unrecognizable letters	Score indicates number of letters that are impossible to identify if considered separately from the text
9	Letter collisions	Number of letters colliding and/or partially overlapping with adjacent ones
10	Max amplitude of letter misalignment	Vertical variance of single letters is measured using graph paper aligned with the ruled paper line. The maximum vertical distance in millimeters above and below the ruled paper line is summed up to obtain an overall score
11	Max variation in size of letters without extension	Height of the smallest and of the biggest letters without extension is measured using graph paper. Score is equivalent to the difference between the two
12	Max variation in size of of letters with extension	Height of the smallest and of the biggest letters with extension is measured using graph paper. Score is equivalent to the difference between the two

### Analyses

For each test we calculated the number of children receiving an ‘adequate’ or ‘inadequate’ outcome. The BVSCO and the BHK tests both map a child’s performance on two nominal levels (‘adequate’, ‘inadequate’), while the DGM-P test provides a three-level outcome (‘adequate’, ‘at risk’, ‘inadequate’). In order to answer our first research question and effectively compare agreement between tests, children that obtained an ‘at risk’ or ‘adequate’ overall performance in the DGM-P test were considered as a unique group and labelled as ‘adequate’. This choice was dictated by the fact that the DGM-P only provides an evaluation of legibility not of dysgraphia (i.e., children that obtain an OPS corresponding to ‘at risk’ outcome in the DGM-P test are not to be classified as severe cases and/or dysgraphic, as children receiving an ‘inadequate’ assessment in the BHK test) (Borean, 2012).

Reliability was assessed by having a second coder (first author), code 26% of sample participants (10 children) for each test. Based on Landis and Koch (1977) level of agreement was classified as follows: 0.00–0.20 slight; 0.21–0.40 fair; 0.4–0.60 moderate; 0.61–0.80 substantial and 0.81–1.00 almost perfect. Following this procedure, inter-coder agreement was significant on the BVSCO test (K_c_ = 1.00, *p* = 0.002, almost perfect agreement), the BHK test (K_c_ = 1.00, *p* = 0.002, almost perfect agreement) and the DGM-P test (K_c_ = 0.583, *p* = 0.006, moderate agreement).

Agreement between tests was evaluated relying on Cohen’s kappa statistics (Watson & Petrie, 2010). This method requires nominal variables with only two mutually exclusive categories, and returns as output: (i) a coefficient on the level of agreement (the k coefficient) (Cohen, 1960); (ii) the *p* value for statistical significance; (iii) a square contingency table reporting frequency distribution of different categories. Values on the main diagonal of the contingency table report the number of times in which two tests provide the same categorical outcome for the same child, while off diagonal elements report the number of times in which tests disagree. Three different Cohen’s Kappa tests were carried out on the entire sample comparing the BVSCO vs. the BHK tests, the BVSCO vs. the DGM-P tests, and the BHK vs. DGM-P tests respectively. The same analyses were then repeated considering respectively only children in second grade and only children in third grade in our sample.

To answer our second research question, analyses on the entire sample were carried out evaluating presence/absence of significant item-rest correlations (also know as corrected item-total correlation) between each GMP (i.e., item) and children’s OPS, in this case computed as the sum of the remaining GMPs (i.e., rest) respectively for the BHK and the DGM-P tests. For example, in the BHK for each child using Pearson’s r we correlated the score obtained on the GMP handwriting size (i.e., item) with the child’s OPS computed as sum of all remaining GMPs minus the GMP handwriting size (i.e., rest). The same procedure was used for all GMPs. Similarly, in the DGM-P: nominal outcomes (‘adequate’, ‘at risk’, ‘inadequate’) on each GMP were transformed into numeric values (‘adequate’ = 0; ‘at risk’ = 0.25; ‘inadequate’ = 1) to obtain an overall numerical OPS as well as individual numerical values for GMPs. Then, for each child, using Pearson’s r we correlated the score obtained on the each GMP (i.e., item) with the child’s OPS computed as sum of all remaining GMPs (i.e., rest). Given that the BVSCO test only provides 1 GMP it was excluded from this analysis.

Finally, a multinomial test on the number of children performing inadequately on each GMP was carried out to explore whether children’s difficulties were equally distributed across all GMPs or if specific GMPs led to greater number of ‘inadequate’ performances in the BHK and DGM-P tests. To carry out this analysis we transformed for each test all scores on individual GMPs into a binary measure (i.e., ‘adequate’, ‘inadequate’). For the BHK test numerical scores on individual GMPs were transformed into binary values (i.e., all GMPs receiving a score > 3 were considered ‘inadequate’, while all scores ≤ 3 were considered ‘adequate’). For the DGM-P test GMPs receiving a nominal value ‘at risk’ or ‘adequate’ were grouped together and labelled ‘adequate’ (as in the between-tests agreement analyses described above), while all others were considered ‘inadequate’.

## Results

Data showed that percentage of children with ‘inadequate’ overall handwriting performance were 35.90% in the BVSCO test, 33.33% in the BHK test and 76.92% in the DGM-P test. Analyses of between-test agreement on overall handwriting performance for our entire sample showed a significant agreement between the BVSCO and the BHK tests (K_c_ = 0.377, *p* = 0.018, fair agreement) and between the BHK and the DGM-P tests (K_c_ = 0.261, *p* = 0.016, fair agreement), but not between the BVSCO and the DGM-P tests (K_c_ = 0.199, *p* = 0.077) (see Table 5). Analyses of agreement between tests on overall handwriting performance for all second graders in our sample showed no significant agreement between tests (i.e., BVSCO vs. BHK: K_c_ = 0.128, *p* = 0.570; BHK vs. DGM-P: K_c_ = 0.174, *p* = 0.179; BVSCO vs. DGM-P: K_c_ = 0.129, p = 0.259) (see Table 6). Considering all third graders in our sample, significant agreement was found only between the BHK and the DGM-P tests (K_c_ = 0.340, *p* = 0.043, fair agreement), while in all other cases agreement did not reach significance (i.e., BVSCO vs. BHK: K_c_ = 0.406, *p* = 0.064; BVSCO vs. DGM-P: K_c_ = 0.255, *p* = 0.178) (see Table 6).

**Table 5:**
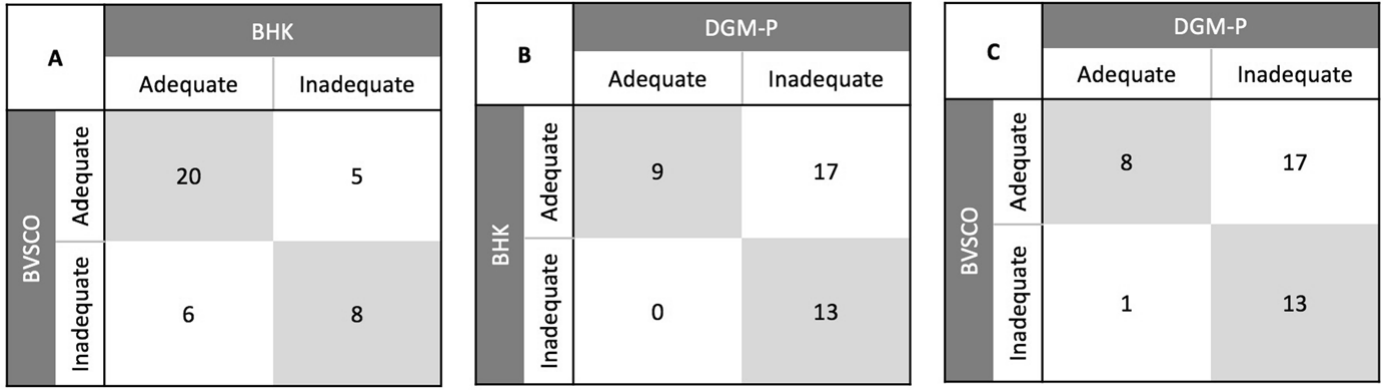
Contingency tables showing agreement between tests on overall handwriting performance on the entire sample, i.e. number of times in which two tests provide the same adequate or inadequate outcome for the same child; comparing respectively: A. BVSCO vs. BHK; B. BHK vs DGM-P; C. BVSCO vs DGM-P

**Table 6:**
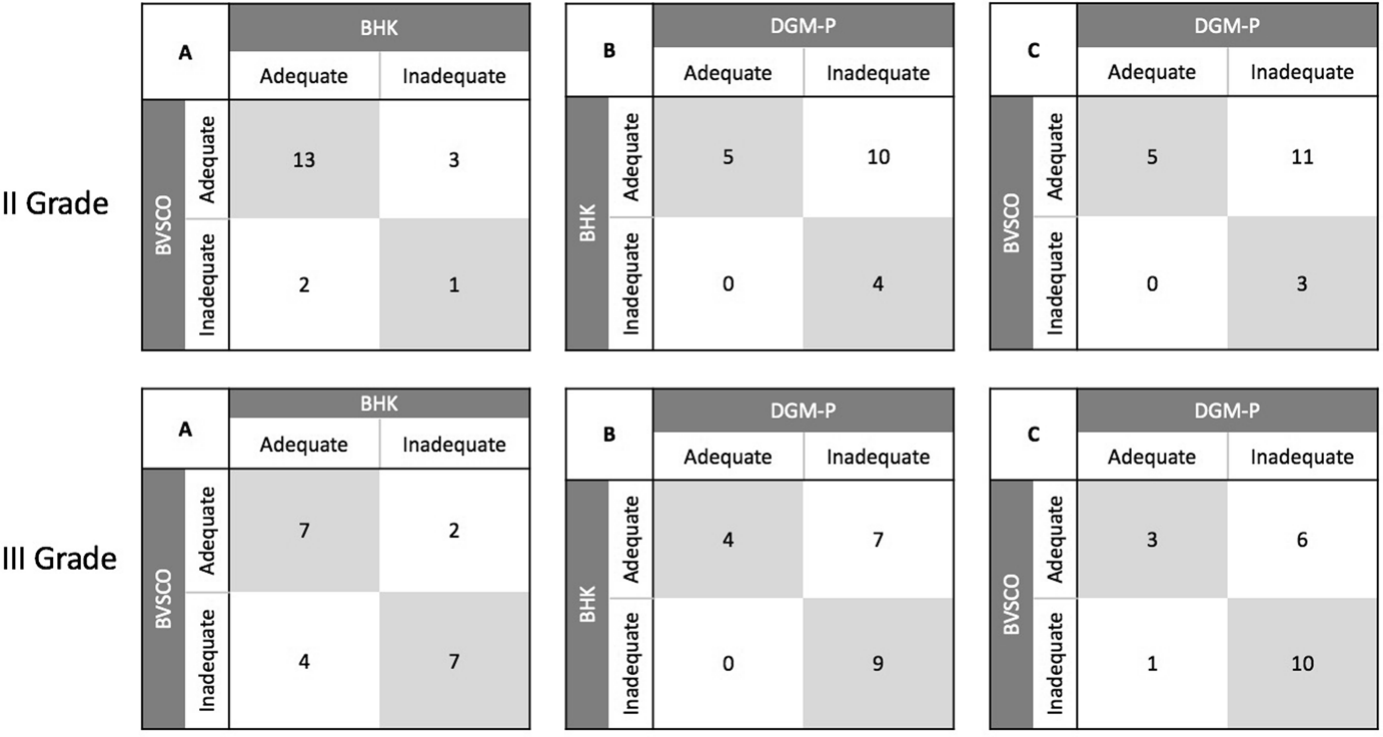
Contingency tables showing agreement between tests on overall handwriting performance in second grader (upper panel) and third grader (lower panel) in our sample, i.e. number of times in which two tests provide the same adequate or inadequate outcome for the same child; comparing respectively: A. BVSCO vs. BHK; B. BHK vs DGM-P; C. BVSCO vs DGM-P

Pearson’s r evaluating presence/absence of significant item-rest correlations on our entire sample, showed significant positive correlations within the BHK for handwriting size (*r* = 0.411, *p* = 0.009), sentence alignment (*r* = 0.379, *p* = 0.017), irregular letter size (*r* = 0.445, *p* = 0.005), incoherent letter size (*r* = 0.537, *p* < 0.001), letter distortions (*r* = 0.667, *p* < 0.001), ambiguous letters (*r* = 0.583, *p* < 0.001) and unsteady trace (*r* = 0.486, *p* = 0.002). While, within the DGM-P test, significant positive correlations emerged for self-corrections (*r* = 0.456, *p* = 0.003), letter alignment (*r* = 0.497, *p* = 0.001), unrecognizable letters (*r* = 0.479, *p* = 0.002), max amplitude of letter misalignment (*r* = 0.535, *p* < 0.001) and max variation in size of medium letters (*r* = 0.519, *p* = 0.001) (see Table 7).

**Table 7:** Results of item-rest correlations using Pearson r for the BHK and the DGM-P tests

	GMPs	*r*
BHK	*Handwriting size*	.411**
	*Margin alignment*	− .093
	*Sentence alignment*	.379*
	*Word spacing*	− .027
	*Acute/long joins*	.298
	*Interrupted/overlapping joins*	.283
	*Letter collisions*	− .029
	*Irregular letter size*	.445**
	*Incoherent letter size*	.537***
	*Letter distortions*	.667***
	*Ambiguous letters*	.583***
	*Self-corrections*	.216
	*Unsteady trace*	.486**
DGM-P	*Speed*	.232
	*Letter forming*	.212
	*Self-corrections*	.456**
	*Letter alignment*	.497**
	*Letter distortions, interrupted/overlapping joins*	.266
	*Ambiguous letters*	.255
	*Incorrect letter size*	.320
	*Unrecognizable letters*	.479**
	*Letter collisions*	− .168
	*Max amplitude of letter misalignment*	.535***
	*Max variation in size of medium letters*	.519**
	*Max variation in size of ascending/descending letters*	.017

^*^p < 0.05; **p < 0.01; ***p < 0.001

**Fig. 2:**
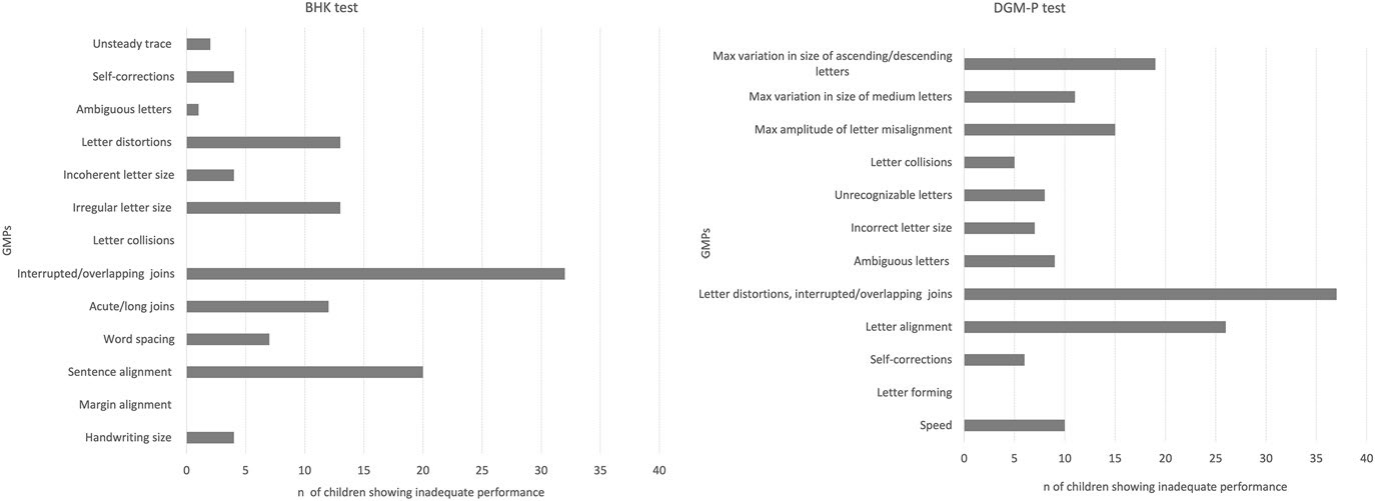
Distribution of number children that showed inadequate performance on individual GMPs in the BHK and the DGM-P tests, considering our entire sample

Multinomial test on number of children with ‘inadequate’ performance on individual GMPs, shows an unequal distribution of ‘inadequate’ assessment in both the BHK and the DGM-P tests, respectively ${\chi }^{2}\left(12\right)=121.071, p<0.001$ and ${\chi }^{2}\left(11\right)=90.686, p<0.001$. Figure 2 shows distribution of children with ‘inadequate’ performance on individual GMPs in our entire sample, allowing to highlight that the two GMPs leading to more occurrences of ‘inadequate’ performance were respectively interrupted/overlapping joins and sentence alignment in the BHK and letter distortions, interrupted/overlapping joins and letter alignment in the DGM-P test. In all these GMPs more than half of our sample received an ‘inadequate’ performance assessment.

## Discussion

Grapho-motor skills involved in handwriting impact both form and content of children’s written texts (Graham et al., 2000; Santangelo & Graham, 2016). Primary school children spend a consistent amount of their school-day in handwriting tasks, but many of them struggle with the production of legible texts, often resulting in poor handwriting or dysgraphia (Marquardt et al., 2016; MI–DGSIS, 2022). Tools to assess and monitor grapho-motor skills in primary school are extremely useful to detect such cases, as well as pinpointing specific grapho-motor parameters (GMPs) that may be hard to tackle for children (Rosenblum et al., 2003). However, little is currently known on between-test agreement, on whether poor performance on specific GMPs is associated with worse overall handwriting performance and on how children’s inadequate performances are distributed across GMPs.

To overcome these limitations our first goal was comparing children’s overall handwriting performance in three gold-standard tests available in Italy (i.e., the BVSCO, BHK and DGM-P tests) to evaluate between-test agreement. Considering our entire participant sample we found significant agreement between the BVSCO and the BHK test and between the BHK and the DGM-P tests, but not between the BVSCO and the DGM-P tests. This result is in contrast with the only previous descriptive study comparing 35 Italian third grade students’ OPS on the BHK and DGM-P tests, which reported scarce agreement between tests, but no statistic data (Neri et al., 2017). However, our findings may be explained considering that these three tests, while sharing what has been termed an “analytic evaluation” of children’s handwriting (Rosenblum et al., 2003, p. 49), rely on very different methods, reaching very different levels of detail. In fact, while the BVSCO measures only one GMP, both the BHK and the DGM-P provide more fine-grained analyses including multiple GMPs. The latter tests however differ in their assessment systems: with the BHK’s scoring one or more lines of text at once and the DGM-P scoring each letter one by one (Borean, 2012; Hamstra-Bletz et al., 1987). In other words, if we were to place the level of detail reached by each of these assessment tools along a continuum, starting from the least detailed to the most fine-grained, the BVSCO and the DGM-P would be at the two extremes of our continuum, with the BHK test somewhere in the middle between them. Therefore, it is not surprising to find that while the BHK shows some level of agreement with the other two tests, tools that rely on extremely different levels of detail end up by showing no significant agreement between them. This result may be of relevance for researchers, who wish to consider both between-test agreement and level of accuracy.

Given that the BVSCO test only measures *speed* of handwriting and that this test shows a fair agreement with the BHK test (i.e., a tests measuring 13 GMPs and in which handwriting speed does not contribute to a child’s handwriting performance), some may suggest that ‘speed’ is a reliable measure of child performance. However, some caution should be exercised in relying exclusively on this measure. Measuring handwriting speed has always been considered relevant in primary school from early studies onwards (Ayres, 1912; Freeman, 1914; Graham et al., 1998) as testified also by numerous tests focusing on this parameter in multiple languages/writing styles (e.g., the Flemish, French and German BHK tests, the Flemish test for writing speed, the Minnesota Handwriting Assessment, the Handwriting Speed Test, the Detailed Assessment of Speed of Handwriting) (Simons & Probst, 2014). However, while multiple studies in different languages have shown that cursive handwriting speed progressively increases with each grade paralleling growth in skill acquisition (Gosse et al., 2021; Hamstra-Bletz & Blöte, 1993; Loizzo et al., 2023; Phelps et al., 1985; Ziviani, 1996), some authors have underscored that this increase may not always be linear (Blöte and Hamstra-Bletz 1991). Graham and colleagues’ (1998) analysis of handwriting speed in children from first to ninth grade in the USA (using print, cursive and mixed handwriting), shows that both girls and boys become gradually faster during the first years of primary school, but plateau between fourth and fifth grade (Graham & Weintraub, 1996; Graham et al., 1998). Similar gradual increase followed by level off after fifth grade is reported in a recent study analyzing cursive handwriting in Turkish children between fourth and eighth grade (Yekeler Gökmen et al., 2022). Previous studies conducted in Italy on primary school students showed that children become faster with each grade, suggesting a linear progression (Accardo et al., 2013; Loizzo et al., 2023; Tressoldi et al., 2019), but these studies only considered children between second and fifth grade. Therefore, we are unable to evaluate presence of a later plateau effect. Given that our sample included second- and third-graders agreement found between the BVSCO and the BHK on overall handwriting performance, may reflect the fact that at this stage of handwriting acquisition speed parallels overall skills acquisition, something that may not hold for older children.

Considering second and third graders separately, we found no agreement between tests in second grade and significant agreement only between the BHK and the DGM-P tests in third grade. This result seems to suggest that for grapho-motor skills to be reliably assessed, children have to have reached at least a basic level of proficiency in cursive handwriting. Children in our sample were introduced to cursive handwriting at the end of first grade and they were evaluated respectively in the second semester of second or third grade. Therefore, second graders in our sample may have not reached sufficient expertise in cursive handwriting to be effectively captured by standardized tests. While this hypothesis may be of relevance for clinicians evaluating grapho-motor skills in primary school children, further data and possibly larger samples are needed to confirm this hypothesis. Taken together, data from between test comparisons seem to suggest that the BHK may prove to be a reliable measure for grapho-motor skills, as long as children have had enough time to practice cursive handwriting.

Our second goal was to investigate whether poor performance on specific GMPs was associated with worse  overall handwriting performance in our sample in order to pinpointing which GMPs may be mainly and significantly associated with a child’s overall score in each test. We expected some characteristics of handwriting would emerge as more relevant towards children’s overall performance, and our results confirmed this hypothesis by showing positive correlations between multiple GMPs and OPS within both the BHK and the DGM-P tests. Considering positive item-rest correlations emerging in both tests we may observe that: alignment (as measured by sentence alignment in the BHK and letter alignment and max amplitude of letter misalignment in DGM-P) and unusual letter shapes (as measure by letter distortions in the BHK and unrecognizable letters as measured in the DGM-P) show positive correlations with OPS in both tests. Given that our sample included children that experienced some difficulties with cursive handwriting (a characteristic that was due to the sample selection process and later confirmed by percentage of children showing ‘inadequate’ OPS), our results may prove helpful by pinpointing the importance of including these aspects of cursive handwriting in future tools for grapho-motor skills assessment. In recent years, attempts have been made to exploit novel screen-based technologies for handwriting assessment. Some studies aimed at implementing criteria used by standardized pen-and-paper tests within software environments. For example, Dimauro and colleagues proposed a software system to support clinicians in diagnosing and monitoring children with dysgraphia called TestGraphia, which implements criteria from the BHK test in a software environment, to support automatic evaluation of specific GMPs starting from a child’s paper text (Dimauro et al., 2020). In other cases, screen-based technologies have been used to directly acquire handwriting data from children with/without dysgraphia and then parse out GMPs which may be of relevance for diagnostic purposes (Asselborn et al., 2018; Mekyska et al., 2017). However promising the use of new technologies may be in assessing handwriting skills, these studies have often shown contrasting results as to which GMPs may be of relevance, in discriminating proficient from non-proficient handwriting (Asselborn et al., 2018; Capellini et al., 2020; Dui et al., 2021; Falk et al., 2011; Giammarco et al., 2016; Mekyska et al., 2017). Our data highlights two GMPs which may prove of relevance for future research in this field, by suggesting that both alignment and unusual letter shapes should be included as relevant GMPs towards OPS.

Finally, results from our exploration of the distribution of children with inadequate performance on specific GMPs in our sample allowed to outline that this distribution was not homogeneous and that some GMPs may lead to a greater number of children with inadequate performance than others. In particular, joins and alignment proved hard to tackle for children in our sample. These data are interesting for two main reasons. First, they show a partially overlapping profile emerging considering individual GMPs assessed by the BHK and the DGM-P test, notwithstanding differences in written samples and scoring methods. Secondly, results from this limited sample suggest that second- and third-graders may find specific aspects of handwriting hard to master (e.g., joins, alignment) in cursive handwriting. Even if in Italy there are no specific guidelines on when to introduce joined cursive handwriting, it is common practice to introduce it quite early (as in France, Germany and the Netherlands), contrary to what commonly happens in other countries (e.g., UK, USA) (Blason et al., 2004; Cotton, 1991; Sassoon, 2003). This is also done because joined cursive handwriting becomes particularly important by the beginning of third-grade, when children are required by national guidelines to start practicing handwriting under dictation (Ministero dell’Istruzione, dell’Università e della Ricerca, 2012). Based on teacher reports, children in our sample had been introduced to joined cursive handwriting at the end of the first-grade and had just begun practicing handwriting under dictation. Therefore, their struggling with joins, while not ascribable to lack of exposure in our sample, was relevant as it may have had an impact on their performance in other school tasks. Some authors suggest that errors in joins may be due to graphic characteristics of words, given that some words require *reshaping joins*, i.e. joins that lead to reshape the way a letter and/or its joins are usually written (Gosse et al., 2021). Such is the case when a *b* is followed by an *r*, which requires to modify both the usual shape and the habitual join of the *r* (Gosse et al., 2021). However, we are not inclined to state that this was the case with our data. In fact, not only children in our sample were required to write texts of varying graphic complexity in the two tests, but texts used in the BHK and the DGM-P only contain a limited number of *reshaping joins* (e.g., a *b* followed by an *e* in the DGM-P test). We are inclined to hypothesize instead that joins are one of the major difficulties posed by cursive handwriting, as the waveform movements of joins require advanced fine-motor control and planning. This is an important aspect that needs to be further investigated in future studies considering larger samples, but our data suggests, to educators and clinicians alike, that greater amounts of practice on joins may be needed to avoid impact of this GMP on texts overall legibility.

We also found that children struggled with handwriting alignment and, relevantly, this is one of the GMPs showing positive correlation with children’s OPSs. These results should be investigated in future studies to better understand whether lack of letter alignment may constitute a useful GMP in detecting poor writers or cases of dysgraphia. We found some limited evidence in support of this hypothesis in a previous study detecting poor letter alignment in children with dysgraphia (Cardoso & Capellini, 2016) and in the widespread practice of using notebooks with highlighted lines to delimit writing space for children that experience handwriting difficulties (Pellegrini & Dongilli, 2010; Pratelli, 2017), but further research is needed.

Overall, our results show that while tests assessing grapho-motor skills in primary school children show some level of agreement, they allow, more importantly, to detect the impact of individual GMPs on children’s performance. In fact, irrespective of differences in measurement and coding methods between these analytic tools, all children in our sample proved to struggle with letter joins and alignment in cursive handwriting. This study is among the few to consider difference in both overall performance and individual GMP scores. However, this work also has some major limitations. First, sample size was limited and confined to primary school children in second and third grade. Second, the current study does not provide fine-grained data on the teaching methods used by teachers’ in our sample, which may impact children’s overall handwriting as well as individual GMPs performance. For example, teachers that have trained on Montessori methods for cursive handwriting often rely on teaching materials (e.g., Metal Insets, Sandpaper Letters) that scaffold the execution and planning of continuous flowing movements required by cursive handwriting, in particular, for the correct execution of letter joins (Lillard, 2017, p. 26). Further studies may also consider the impact of specific teaching methods on children’s grapho-motor skills. Notwithstanding these limitations, we hope that data provided in this study may be put to good use by teachers, clinicians and therapists when choosing among tests for grapho-motor skills assessment and in aiding the acquisition of specific GMPs in primary school children.

## Footnotes

1 Generalizations on the most appropriate time for cursive assessment should be avoided, given that correct age may vary between countries or even between schools within the same country due to educational approaches. In fact, appropriate timing for cursive assessment largely depends on when and if children are actually introduced to using this handwriting style and begin consistently using it, so as to consolidate the sensory-motor patterns involved in handwriting. For example, in Italy most public schools begin teaching cursive handwriting by the end of first grade or at the beginning of second grade. These skills are progressively consolidated in the second semester of second grade and the first semester of third grade. However, in Montessori schools (e.g., public schools explicitly adopting the Montessori method) children begin exercising sensory-motor patterns that support cursive handwriting from kindergarten and begin consistently using cursive from the beginning of first grade also relying on a variety of dedicated materials (Lillard, 2017). Therefore, children’s competencies in cursive handwriting may vary in some countries even between schools, with a relevant impact on appropriate timing for grapho-motor skills assessments.

2 Some authors have argued that function (i.e., readability) rather than topography should constitute the basis of handwriting assessments (Talbert-Johnson et al., 1991), but analytic tools are still considered the gold standard for handwriting assessments due to greater consistency and higher inter-rater agreement compared to other methods (Rosenblum et al., 2003).

3 For example, according to test normative data mean number of graphemes produced by second graders in the third trimester is 60 (standard deviation 12), while mean number of graphemes produced by third graders in the third trimester is 71 (standard deviation 13).

4 Following test normative data mean OPS in second grade and third grade is 20.3 (standard deviation 6.1) for males and 18.1 (standard deviation 6.7) for females.
